# Only Surgical Treatment to Be Considered for Adhesive Small Bowel Obstruction: A New Paradigm

**DOI:** 10.1155/2018/9628490

**Published:** 2018-10-23

**Authors:** Nicolas Tabchouri, David Dussart, Urs Giger-Pabst, Nicolas Michot, Frederic Marques, Meriem Khalfallah, Petru Bucur, Louise Barbier, Aurore Kraemer-Bucur, Mihane Nayeri, Julien Thiery, Celine Bourbao-Tournois, Pascal Bourlier, Ephrem Salamé, Mehdi Ouaïssi

**Affiliations:** ^1^Department of Digestive, Oncological, Endocrine, Hepato-Biliary and Pancreatic Surgery, and Liver Transplantation, Trousseau Hospital, University Hospital of Tours, France; ^2^Department of General Surgery & Therapy Center for Peritoneal Carcinomatosis, St. Mary's Hospital, University Hospital of the Ruhr-University Bochum, Herne, Germany

## Abstract

**Background:**

Adhesive small bowel obstruction (SBO) represents a heavy burden in healthcare systems worldwide and is associated with significant morbidity and mortality. Although conservative treatment alone can lead to SBO resolution in most cases, its optimal duration is still a matter of debate. The aim of this study was to analyze different SBO evolution patterns in order to further determine when to switch to surgical treatment.

**Study Design:**

All patients who were admitted for adhesive SBO between 2011 and 2016 were reviewed. Patients who had immediate surgery (IS), a successful medical treatment (SMT), and a failed medical treatment (FMT) were compared in terms of overall morbidity, mortality, and SBO recurrence.

**Results:**

Overall 154 patients were identified, including 23 (14.9%) in IS, 27 (17.5%) in FMT, and 104 (67.6%) in SMT groups. In terms of comorbidities, patients were similar in all groups. Overall morbidity rates were highest in IS and FMT groups (30% and 33%, respectively, vs. 4% in the SMT group, *p* < 0.001) whereas mortality rate was highest in the FMT group (22% vs. 0% and 0% in IS and SMT groups, respectively, *p* < 0.001). SBO recurrence rate was highest in the SMT group (22% vs. 4% and 7% in IS and FMT groups, respectively, *p* = 0.042).

**Conclusion:**

FMT seems to be associated with similar overall morbidity compared with IS but with increased postoperative mortality. Patient frailty seems to be worsened by prolonged inefficient medical treatment.

## 1. Introduction

Peritoneal adhesions are the underlying cause of 32% of acute intestinal obstructions and of 65%–75% of small bowel obstructions (SBO) and represent a major unresolved public health issue and burden [1]. In patients with abdominal pain, SBO is a common cause that accounts for 4% of all emergency department admissions and 20% of emergency surgical procedures [2]. Mortality rates of patients surgically treated for SBO remain surprisingly high (5–10%) [1, 3–5].

Conservative SBO treatment was acknowledged by published reports and clinical practice when patients did not present any sign of strangulation, peritonitis, or severe intestinal impairment and when computed tomography (CT scan) revealed no small bowel feces sign, free intraperitoneal fluid, or mesenteric edema [6]. However, no consensus has been reached regarding conservative treatment duration or when to switch to operative treatment in case of failure. Despite two large cohort studies demonstrating that mortality rates were increased in SBO patients undergoing surgery with a 24-hour delay [5, 7], the 2013 World Society of Emergency Surgery recommendations state that surgical treatment should be considered in the absence of SBO resolution after a 72-hour nonoperative management duration [6]. These conflicting results are due to the usually heterogeneous nature of patients' inclusion. Very few data were reported in the literature about short- or long-term outcome of patients which have had a failure of medical treatment of SBO. The present study aimed at determining results of different surgical managements of SBO in the same surgical team with homogeneous management with a focus on the specificity of the group of patients which have a failure of their medical treatment.

## 2. Methods

### 2.1. Study Population and Data Collection

From January 2011 to May 2016, all consecutive patients treated for adhesive SBO at the University Hospital of Tours were identified and retrospectively included. During the study period, the first admission for SBO was defined as the index date. Other causes for SBO including malignancy, volvulus, postoperative ileus (within 1 month), inflammatory bowel disease, large bowel obstruction, hernia, radiation-related obstruction, and Meckel's diverticulum were excluded [8–11]. At initial admission, CT scan and blood work were performed for all patients.

### 2.2. SBO Management

SBO surgical treatment upon admission (immediate surgery (IS)) was decided based on clinical evaluation (spontaneous and/or provoked abdominal pain, abdominal guarding, hemodynamic choc, signs of strangulation, peritonitis, and fever) and imaging severity signs (such as free intraperitoneal fluid, mesenteric edema, lack of small bowel feces sign, bowel strangulation, devascularized bowel, necrosis, and perforation) [6]. When severity signs were absent, patients were treated medically (with intravenous hydration, fasting, nasogastric tube drainage, and analgesics) and were clinically and biologically reevaluated every 6 hours. Failed medical treatment (FMT) was diagnosed if severity signs appeared or if SBO resolution was not reached and led to secondary surgical treatment (the decision to switch to surgical treatment was left at each physician's discretion). Patients treated medically and who died before undergoing surgery were accounted for as FMT patients. Each patient was admitted and reevaluated by the same senior surgeon. Patients were therefore divided into three groups: immediate surgery (IS), failed medical treatment (FMT), and successful medical treatment (SMT). These groups, which represented three different SBO profiles and evolution patterns, were then compared.

### 2.3. Study Variables

The following baseline demographic and clinical characteristics were collected: age, gender, BMI, comorbidities, previous surgical procedures, American Society of Anesthesiologist (ASA) score and Charlson comorbidity index (CCI) [12, 13]. Clinical, biological (including C-reactive protein (CRP), electrolyte fluid analysis, and white blood cell (WBC) count), and CT scan evaluation and results upon admission were collected, as well as clinical and biological results at each reevaluation. Medical treatment duration was noted in all FMT patients. The following intraoperative and postoperative variables were also collected: operative duration, adhesion type (single band or extensive intra-abdominal adhesions), small bowel injury occurrence, and whether or not resection was required. We also recorded whether or not surgical treatment was performed during the night (between 6:30 pm and 6:30 am).

### 2.4. Postoperative Outcomes

Postoperative morbidity and mortality were assessed at 30 days following surgery using Clavien-Dindo classification. Severe postoperative complications were defined as Clavien-Dindo > 2 [14]. Anastomotic leakage, intra-abdominal abscess, and postoperative collections were searched for if patient presented with signs of sepsis, using CT scan. Other postoperative complications were also collected such as hemorrhage, anastomosis stenosis, and other infectious and cardiorespiratory complications. Therapeutic management of postoperative complications was also recorded. Postoperative bowel recovery was determined by flatus, passage of stool, and oral intake recovery. In the SMT group, median time to flatus and oral intake recovery was collected. Total in-hospital stay and readmission rates were collected. Readmissions due to SBO recurrence as well as subsequent therapeutic management were specifically recorded. Patients who were not readmitted were contacted by phone in order to assess any SBO recurrence symptoms. Follow-up was updated to May 2016. Further analyses were performed in the FMT group according to medical treatment duration (<48 h and >48 h).

### 2.5. Statistical Analysis

Baseline characteristics of the studied population and intraoperative and postoperative outcomes were analyzed. Categorical variables were compared using the chi-squared test or Fischer's exact test when appropriate. Bonferroni's correction was also used whenever appropriate. Continuous variables were compared using Student's *t*-test or the Mann-Whitney *U* test whenever appropriate. Categorical variables were expressed as numbers (percentages) and continuous variables as means (±standard deviation (SD) or range). All statistical analyses were performed using IBM SPSS Statistics version 20 (IBM SPSS Inc., Chicago, IL, USA), and statistical significance was accepted at the 0.05 level.

## 3. Results

### 3.1. Baseline Characteristics (Table 1)

Overall, 154 patients presenting with adhesive SBO were included in the study period. Median age was 74 (16–104) years and there were 85 men (55.2%). All patients had a previous abdominal surgery by open procedure. Immediate surgery (IS group) was required in 23 (14.9%) patients, and failed medical treatment was observed in 27 (17.5%) patients (FMT group). In 104 (67.6%) patients, medical treatment was sufficient and led to SBO resolution (SMT group). In terms of comorbidities, atrial fibrillation was significantly more frequent in the SMT group (IS, 4.3%; FMT, 7.4%; and SMT, 18.3%; *p* = 0.001) and chronic obstructive pulmonary disease was significantly more frequent in IS (IS, 13%, FMT: 0%, and SMT: 2.9%; *p* = 0.038). Charlson comorbidity index was not significantly different between the three groups. In terms of clinical presentation, spontaneous and provoked abdominal pain was significantly more frequent in the IS group compared to the FMT and SMT groups (95% vs. 40% and 54%, *p* < 0.001 and 96% vs. 93% and 70%, *p* = 0.004, respectively). IS patients presented with significantly more intraperitoneal fluid, feces sign, and devascularized bowel compared with FMT and SMT patients (*p* < 0.001). In terms of biological evaluation, WBC count was significantly increased in the IS group (*p* = 0.040).

### 3.2. Surgical Treatment

In the FMT group, the decision to switch to surgical treatment was taken because of persistence or worsening of abdominal pain (*n* = 7, 26%), lack of SBO resolution after a 48-hour period of time (*n* = 17, 63%), and SBO recurrence after feeding reintroduction during the same in-hospital stay (*n* = 3, 11%). In the FMT group, 2 (7.4%) patients died before reaching the operative room. Surgical treatment consisted of open procedures in all cases. Extensive adhesiolysis and small bowel resection were required in 40% and 18% of all patients who underwent surgical SBO treatment (IS and FMT groups, *n* = 50). Most surgical procedures (IS and FMT groups) were performed during the night (*n* = 30, 60%). In the FMT group, mean medical treatment duration was 4 ± 2 days; in FMT, 33% (*n* = 9) and 67% (*n* = 18) of patients were treated ≤48 h and >48 h, respectively.

### 3.3. Postoperative Morbidity and Mortality

Overall morbidity and mortality rates are presented in Table 2. There was no intraoperative death. Six patients (6.7%) died postoperatively from pancreatitis (*n* = 1), cardiorespiratory complication (*n* = 3), and peritonitis (*n* = 2). Overall postoperative morbidity rate was 12.9% (*n* = 20) and was significantly different between the 3 groups. Ten (6.5%) patients presented with grade I–II postoperative complications according to Clavien-Dindo classification and 10 (6.5%) with grade III–IV complications. In the IS group, one (4.3%) patient presented with an anastomotic leak, one (4.3%) with an intra-abdominal abscess, 4 (17.3%) with a urinary infection, and one (4.4%) with an incisional abscess. Mortality rate was null.

In the FMT group, mortality rate was 22.2% (*n* = 6): one (3.7%) patient died of pancreatitis, 2 (7.4%) before undergoing surgery, one (3.7%) due to cardiorespiratory complication (requiring critical care), and 2 (7.4%) due to anastomotic leak occurrence (redo surgery was not performed in both latter patients due to very advanced age: 95 and 104 years old). In the SMT group, one (0.9%) patient needed intensive care for hemodynamic instability. Two (1.8%) patients presented with a catheter-related skin infection and one (0.9%) with a urinary infection.

The FMT group was further analyzed according to conservative treatment duration (failure less than 48 hours following medical treatment introduction (FMT < 48 h, *n* = 9) and failure more than 48 hours following introduction (FMT > 48 h, *n* = 18)). Observed mortality rates were 33% (*n* = 3) in FMT < 48 h vs. 16.6% (*n* = 3) in FMT > 48 h, *p* = 0.367.

### 3.4. SBO Recurrence

SBO recurrence was observed in 27 (16.9%) patients. In the SMT group, 23 (22.1%) patients presented with SBO recurrence vs. one (4.3%) and 2 (7.4%) in the IS and FMT groups, respectively (*p* = 0.042). SBO recurrence surgical treatment was required in 4 (3.8%) SMT patients vs. *n* = 2 (7.4%) and *n* = 1 (4.3%) patients in the FMT and IS groups, respectively. Median follow-up duration in the entire population was 34 months (2–179) and was not significantly different between the three groups. Median time to SBO recurrence was 38 months (1–172) Table 2.

## 4. Discussion

If adhesive SBO medical treatment is widely accepted as a potential therapeutic option, the exact duration before deciding to switch to a surgical treatment in case of failure has yet to be determined. In clinical practice, SBO management is based on physical examination (mainly pain severity), CT scan results, patient comorbidity, and surgeon's clinical experience. The World Society of Emergency Surgery has published recommendations regarding SBO management in 2013, stating that surgical treatment should be proposed whenever medical treatment failed to achieve SBO resolution 72 hours following introduction [6]. But, most physicians prefer a conservative management in SBO patients presenting with important comorbidities, believing this will decrease mortality rates compared with surgical treatment. Indeed, surgeons do not seem to strictly abide by these recommendations and medical treatment duration can go up to 9 days according to some [3, 15]. The present study sought to analyze different adhesive SBO profiles and their respective outcomes in order to further improve therapeutic management.

In this series, IS patients presented with significantly more grade III–IV postoperative complications compared with FMT patients (9% vs. 4%, *p* = 0.010), but mortality rates were highest in FMT patients (0% vs. 22%, *p* = 0.042). Although age and comorbidities were not different between the groups, the occurrence of grade III–IV postoperative complications seemed to lead to impaired survival in patients who previously underwent medical treatment (FMT) compared with those who immediately underwent surgery (IS). A prolonged medical treatment might therefore be responsible of increasing patient frailty and decreasing their resistance in case of postoperative complications' occurrence.

Common arguments against early surgical treatment when confronted with adhesive SBO include worsened outcome compared with successful medical treatment and an increased SBO recurrence rate [7, 16]. Current analysis was not in accordance with these arguments, showing that grade III–IV–V postoperative complications were higher in initially medically treated patients, and SBO recurrence rate was highest in the SMT group. Indeed, although most published studies have reported increased postoperative complications in operatively managed SBO patients compared with medically managed patients, one possible explanation may be that surgically treated FMT patients should be taken into account differently than IS patients [9]. Because of profile difference between IS and FMT patients, we chose to separately analyze their outcomes. When comparing IS and SMT patients, the present results are in accordance with other published reports. Compared with IS patients, SMT patients presented with less severe postoperative complications but postoperative mortality was similar [7, 16]. Whereas regardless of medical treatment duration, FMT patients presented with worse outcomes (morbidity and mortality) compared with IS patients. Weaknesses of the study include its retrospective design and its relatively low number of cases (compared with other published reports) [7, 9]. However, strengths include the fact that patients were divided into 3 groups (IS, FMT, and SMT) unlike most authors who analyzed results according to 2 groups (medical and surgical treatment). Indeed, separating patients into 3 groups has led to identify patients with considerably impaired outcome (FMT).

The present results revealed a lower recurrence rate in SBO patients who were surgically treated (IS + FMT, *n* = 3, 6%) compared with SMT patients (*n* = 23, 22.1%, *p* = 0.042). This is in accordance with other published series. The alleged “paradox” arising from “surgical treatment induced adhesions” should no longer be considered a valid argument in choosing therapeutic management [1].

## 5. Conclusions

FMT seems to be associated with similar overall morbidity compared with IS but with increased postoperative mortality and recurrence rates of SBO. Patient frailty seems to be worsened by prolonged inefficient medical treatment. This study showed that SBO should be treated earlier by surgery in order to decrease mortality rates and recurrences of SBO.

## Figures and Tables

**Table 1:** Demographic characteristics and initial SBO clinical, radiological, and biological presentations.

	IS (*n* = 23)	FMT (*n* = 27)	SMT (*n* = 104)	Total (*n* = 154)	*P*
*Demographic characteristics*					
Age (years)	69 (25–97)	70 (22–104)	76 (16–99)	74 (16–104)	0.115
Gender (M/F)	9/14	12/15	65/39	85/69	0.052
BMI (kg/m^2^)	23.5 (16.9–81)	20.3 (16.5–30.9)	23.1 (14.8–40.1)	23.8 (14.8–40.1)	**0.004**
ASA score, *n* (%)					
1–2	19 (82.6)	19 (70.4)	83 (79.8)	121 (78.6)	
3–4	4 (17.4)	8 (29.6)	21 (20.2)	33 (21.4)	0.497
Previous SBO, *n* (%)	14 (60.9)	4 (14.8)	41 (39.4)	59 (38.4)	**0.004**
Comorbidity, *n* (%)					
Diabetes mellitus, *n* (%)	0	2 (7.4)	17 (16.4)	19 (12.3)	0.068
Renal failure, *n* (%)	1 (4.3)	2 (7.4)	13 (12.5)	20 (12.9)	0.437
Peripheral arterial disease, *n* (%)	4 (17.3)	3 (11.1)	19 (18.3)	26 (16.9)	0.788
Coronary disease, *n* (%)	2 (8.7)	2 (7.4)	15 (14.4)	19 (12.3)	0.520
Atrial fibrillation, *n* (%)	1 (4.3)	2 (7.4)	34 (32.7)	37 (24.1)	**0.001**
Previous stroke, *n* (%)	2 (8.7)	2 (7.4)	7 (6.7)	11 (7.1)	0.113
Elevated blood pressure, *n* (%)	12 (52.2)	6 (22.2)	45 (43.3)	63 (40.9)	0.069
COPD, *n* (%)	3 (13.0)	0	3 (2.9)	6 (3.9)	**0.038**
Performance status, *n* (%)					
(i) 0	6 (26.1)	8 (29.6)	23 (22.1)	37 (24.1)	0.542
(ii) 1	8 (34.8)	9 (33.3)	46 (44.3)	63 (40.9)	
(iii) 2	8 (34.8)	5 (18.5)	23 (22.1)	36 (23.4)	
(iv) 3	1 (4.3)	3 (11.1)	10 (9.6)	14 (9.1)	
(v) 4	0	2 (7.4)	2 (1.9)	4 (2.6)	
Charlson comorbidity index, *n* (%)					
(i) 0	13 (56.5)	13 (48.1)	41 (39.4)	67 (43.5)	0.243
(ii) 1–2	9 (39.1)	9 (33.3)	37 (35.6)	55 (35.7)	
(iii) 3–4	1 (4.3)	3 (11.1)	23 (22.1)	27 (17.5)	
(iv) ≥5	0	2 (7.4)	3 (2.8)	5 (3.2)	
*Clinical presentation*					
Spontaneous abdominal pain, *n* (%)	22 (95.6)	11 (40.1)	56 (53.9)	89 (57.8)	**<0.001**
Provoked abdominal pain, *n* (%)	22 (95.6)	25 (92.6)	73 (70.2)	120 (77.9)	**0.004**
*Radiological presentation*					
Free peritoneal fluid, *n* (%)	13 (56.5)	8 (29.6)	13 (12.5)	34 (22.1)	**<0.001**
Feces sign, *n* (%)	19 (82.6)	3 (11.1)	20 (19.2)	42 (27.3)	**<0.001**
Devascularized bowel, *n* (%)	19 (82.6)	1 (3.7)	2 (1.9)	22 (14.3)	**<0.001**
*Biological presentation*					
WBC count (G/L)	13.5 (8.3–18.9)	10.2 (5.3–15.6)	10.8 (4.9–17.1)	11.1 (6.2–17.5)	**0.042**
CRP (mg/L)	32	39.8	26.7	29.8	0.304

SBO: small bowel obstruction; IS: immediate surgery; FMT: failed medical treatment; SMT: successful medical treatment; BMI: body mass index; ASA: American Society of Anesthesiologists; COPD: chronic obstructive pulmonary disease; WBC: white blood cells; CRP: C-reactive protein. Continuous variables are presented as mean (range).

**Table 2:** Outcome according to different SBO therapeutic managements.

	IS (*n* = 23)	FMT (*n* = 27)	SMT (*n* = 104)	Total (*n* = 154)	*P*
Single band adhesion, *n* (%)	13 (56.5)	17 (63.0)	—	30 (60.0)	0.774
Extensive adhesions, *n* (%)	10 (43.5)	10 (37.0)	—	20 (40.0)	0.640
Associated bowel resection, *n* (%)	5 (21.7)	4 (14.8)	—	9 (18.0)	0.715
Night shift surgery, *n* (%)	17 (73.9)	13 (48.1)	—	30 (60.0)	1.000
Operative time (min)	120 (70–180)	117 (70–160)	—	120 (70–172)	0.871
*Short-term outcome*					
Overall complications, *n* (%)	7 (30.4)	9 (33.3)	4 (3.8)	31 (20.1)	**<0.001**
Grade I–II, *n* (%)	5 (21.7)	2 (7.4)	3 (2.9)	10 (6.5)	**0.004**
Grade III–IV, *n* (%)	2 (8.7)	1 (3.7)	1 (0.9)	4 (3.8)	**0.010**
Reoperation, *n* (%)	0	1 (3.7)	0	1 (0.65)	—
Mortality, *n* (%)	0	6 (22.2)	0	6 (3.9)	**<0.001**
Anastomotic leak, *n* (%)	1 (4.3)	3 (11.1)	0	4 (2.6)	**0.005**
Peritonitis, *n* (%)	0	2 (7.4)	0	2 (1.3)	**0.009**
Intra-abdominal collection, *n* (%)	1 (4.3)	1 (3.7)	0	2 (1.3)	0.119
Superficial abscess, *n* (%)	1 (4.3)	1 (3.7)	0	2 (1.3)	0.119
Cardiac failure, *n* (%)	0	0	0	0	1.000
Pulmonary complication, *n* (%)	0	2 (7.4)	0	2 (1.3)	**0.009**
Urinary infection, *n* (%)	4 (17.4)	4 (14.8)	1 (0.9)	9 (5.8)	**0.001**
*Long-term outcome*					
Follow-up (months)	47 (2–147)	34 (2–57)	34 (2–179)	34.7 (2–179)	0.648
SBO recurrence, *n* (%)	1 (4.3)	2 (7.4)	23 (22.1)	26 (16.9)	**0.042**
Stoma requirement, *n* (%)	1 (4.3)	2 (7.4)	0	3 (1.9)	**0.031**
Anastomotic stenosis, *n* (%)	1 (4.3)	0	0	1 (0.6)	0.057

SBO: small bowel obstruction; IS: immediate surgery; FMT: failed medical treatment; SMT: successful medical treatment. Continuous variables are presented as mean (range).

## Data Availability

All data were retrieved from patient files from the Department of Digestive Surgery at the University Hospital of Tours. All data used to support the findings of this study were rendered anonymous and are available from the corresponding author upon request.
